# Analysis of the Effect of Wearing Extensible and Non-Extensible Lumbar Belts on Biomechanical Factors of the Sit-to-Stand Movement and Pain-Related Psychological Factors Affecting Office Workers with Low Back Pain

**DOI:** 10.3390/healthcare9111601

**Published:** 2021-11-22

**Authors:** Sang-Cheol Im, Ho-Young Cho, Jae-Hong Lee, Kyoung Kim

**Affiliations:** 1 Department of Physical Therapy, College of Rehabilitation Science, Daegu University, Gyeongsan 38453, Korea; marinept83@daum.net (S.-C.I.); welove24@naver.com (H.-Y.C.); 2 Department of Physical Therapy, Daegu Health College, Daegu 41453, Korea; heart0630@dhc.ac.kr

**Keywords:** low back pain, sit-to-stand, lumbar belt, biomechanics, pain-related psychology

## Abstract

This study aimed to investigate the effects of wearing extensible and non-extensible lumbar belt (LB) on biomechanical factors of the sit-to-stand (STD) movement and pain-related psychological factors affecting office workers with low back pain. Among 30 office workers, 15 with low back pain (LBP) were assigned to the experimental group and 15 healthy adults were assigned to the control group. The participants performed STD movement in random order of three different conditions: without LB (Condition 1), with extensible LB (Condition 2), and with non-extensible LB (Condition 3). Biomechanical variables of STD movement in each condition were measured using a three-dimensional motion analysis system and force plate. Pain-related psychological factors were measured only in the experimental group. Among the biomechanical factors of STD movement, an interaction effect was found in the maximum anterior pelvic tilt angle and total-phase range of motion of the trunk (*p* < 0.05). Pain intensity, pain-related anxiety, and pain catastrophizing were decreased in the conditions with lumbar belts (Conditions 2 and 3) compared to the condition without LB (Condition 1) (*p* < 0.05). Extensible and non-extensible lumbar belts engender biomechanically beneficial effects during STD movement in both office workers with LBP and healthy office workers. Further, pain intensity, pain-related anxiety, and pain catastrophizing were decreased in office workers with LBP. Therefore, both types of extensible lumbar belts may be helpful in the daily life of patients with LBP and office workers.

## 1. Introduction

Low back pain (LBP) is one of the most common health conditions, which has an enormous socioeconomic impact [1]. It has been confirmed to be the main cause of disorders around the world in a recent Global Burden of Disease study [2]. LBP is the pain emerging on the back of the body, ranging from the lower edge of the 12th rib to the gluteal fold; the pain is severe enough to limit normal activities for more than a day, regardless of radiating pain to lower extremities [3]. Balance and ambulatory abilities of patients with LBP are decreased compared with those of healthy individuals [4], thereby limiting physical function and engendering inabilities resulting from decreased muscle strength, endurance, and coordination [5]. Therefore, absence of labor and loss of productivity are common in patients with LBP because of difficulty to work, absence, etc. [6]. Moreover, as a major reason for health care costs, the economic impact of LBP is similar to that of other general high-cost diseases such as cardiovascular diseases, cancer, mental health disorders, and autoimmune diseases [7]. Eventually, LBP results in extensive economic pressure on individuals, families, communities, industries, and governments [8].

Sit-to-stand (STD) movement is a basic activity of daily life necessary for correct posture, initiation of walking, and personal care [9]; it is the most frequently performed functional activity [10]. For daily activities and functional independence, STD movement and stand-to-sit movement are performed ≥60 times on average in a day, and this movement accounts for 56–64% of total lumbar sagittal mobility [11]. The standard anatomical position of modern humans is the sedentary position considering that modern humans spend most of their time in this position, including their working and free time [12]. Notably, the occurrence rate of LBP has been increased among office workers [13]. In particular, difficulty in performing the STD movement has been reported frequently in patients with LBP [14], and this is associated with worsening symptoms of LBP [15]. Considering that the STD movement is performed constantly in a day, excessive pressure may be exerted on the physical structure, which could be a risk factor for causing LBP that is persistent [16]. Therefore, it is important for patients with LBP to perform the STD movement accurately, which makes studies on the STD movement essential [17].

According to several studies, patients with LBP have impaired proprioceptive sense of the lumbar region [18,19,20]. This impaired perception could cause LBP and may result in or sustain joint instability [21]. Hence, wearing a lumbar belt (LB) may be beneficial in such patients [22]. Wearing a LB increases the pressure exerted on the skin, which provides additional afferent sensory information to the central nervous system via mechanical receptors, which may ultimately improve the proprioceptive sense of the lumbar region [23]. In addition, wearing a LB increases mechanical rigidity, which may decrease the structural load of the spine by limiting lumbar movement [24]. For example, LB can decrease the stress in viscoelastic structures of the back and the compressive loads on the lumbar spine [25,26]. Owing to these advantages, office workers use LB to reduce LBP and waist injury [27]. LB are also used to reduce healthcare costs and improve the functional condition of individuals suffering from LBP [28]. Similarly, LB are frequently used to prevent LBP as a convenient and conservative treatment, with a substantially high satisfaction level [29]. However, the potential effects of using a LB for treating LBP remain unclear owing to the limited evidence in this field and no clear guidelines on LB use for patients with LBP [30]. Therefore, further studies on the biomechanical effects of using LB in patients with LBP are warranted. In addition, regarding pain-related psychological mechanisms, wearing a LB provides immediate pain relief and reduces pain-related anxiety [31]. Although wearing a LB may gradually aid workers, who otherwise have to absent from work because of LBP, in performing physically demanding work and securing their job, studies on the immediate and long-term effects of LB in terms of these pain-related psychological mechanisms are lacking.

Wearing a LB is benefits workers who want to keep their job despite LBP; however, there in insufficient evidence on the efficacy and benefits of wearing a LB. In particular, till date, there have been no studies investigating the effects of a LB on STD movement, which is an important functional activity for office workers who spend much of the time in a sitting position and patients with LBP. Therefore, the first aim of this study was to investigate the effect of wearing extensible and non-extensible LB on biomechanical factors in office workers with LBP while performing the STD movement. The second aim was to investigate the effect of wearing extensible and non-extensible LB on pain-related psychological factors in office workers with LBP while performing the STD movement. We aimed to provide evidence on the efficacy of using LB for patients and office workers with LBP and present basic data for preventing and treating LBP. Our hypotheses were as follows: first, according to the presence or absence of LBP and the conditions of a wearing LB, the impact on the biomechanical factors of the STD movement will differ; second, there may be a difference in the effect on pain-related psychological factors associated with the STD movement in office workers depending on the conditions of a wearing LB.

## 2. Materials and Methods

### 2.1. Participants

Among outpatients and guardians who visited our hospital between April 2020 and May 2021, office workers aged 20–50 years were recruited. Out of 30 participants, 15 patients with nonspecific LBP were assigned to the experimental group and 15 healthy adults were assigned to the control group. Age, sex and body mass index (BMI) were matched between the two groups. Using a previous study with the same design (2 groups × 3 conditions) as a reference, G-power 3.1.9.4 was used to determine the sample size [31]. The effect size of the total lumbar range of motion (ROM) variables of the manual material handling task from the previous study was used. A total of 30 subjects were selected based on the effect size of 0.31, significance level of 0.05, and power of 80% [32].

Inclusion criteria for the experimental group were lumbar or lumbosacral pain without radiating pain below the knee for at least 4 weeks (nonacute). Exclusion criteria for the experimental group included spinal surgery, specific lumbar pathology (facture, infection, tumor, etc.), scoliosis, systemic or degenerative diseases, BMI > 30 kg/m^2^, hypertension (systolic blood pressure >140 mmHg; diastolic blood pressure >90 mmHg), history of nervous system disease, and taking medication that may affect nervous excitability [31]. Exclusion criteria for the control group included history of persistent LBP lasting over a week within the last year [22]. In addition, patients with neurological or respiratory diseases that may affect the STD movement or trauma, or pregnant patients were excluded from both groups [33]. Eligibility for participation was identified using medical records, imaging studies, and interviews.

After explaining the purpose of the study, the contents of the experiment, and the degree of exposure during the experiment to all participants in the experiment, a written informed consent for voluntary participation was obtained. Sex, age, and anthropometric measurement data of all participants were collected, and pain (Visual Analog Scale, VAS) [34] and disability (Oswestry Disability Index, ODI) [35] in the experimental group with nonspecific LBP were additionally investigated. This study was approved by the institutional review board of Daegu University (040621-201911-HR-025-02) and was conducted in accordance with the Declaration of Helsinki. The study was also registered in the Clinical Research Information Service (CRIS) of Republic of Korea for ethical purposes, sharing information, and promoting transparency in clinical research (clinical trial registration number: KCT0004970).

### 2.2. Lumbar Belts

**Figure 1:**
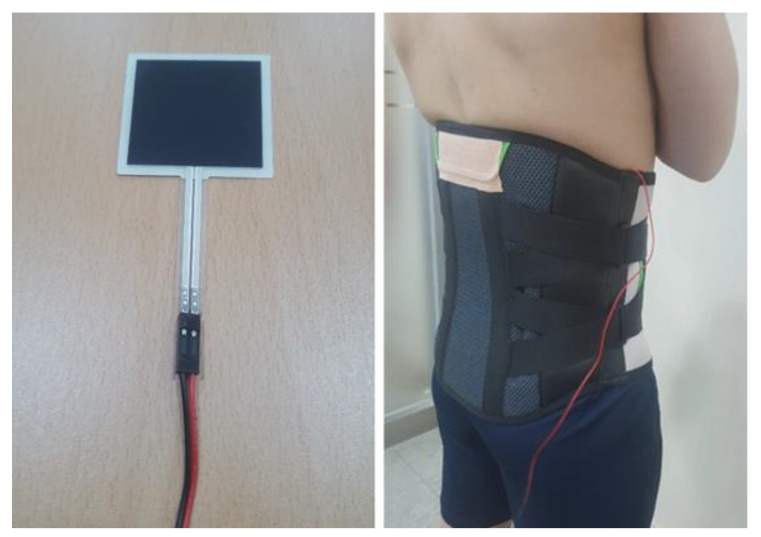
Measurement of the pressure of the lumbar belt using a force-sensing resistor.

Considering flexibility, comfort, and economical efficiency for use in daily activities and at work, two types of LB were chosen after consulting orthopedists. Both types of LB consisted of a two-layered fixable strap made of Velcro. Initial adjustment and placement of the LB was performed using the internal layer, and final tension was adjusted with the external layer. The extensible LB (REDIX-K210, Acetech, Seoul, Korea) was composed of only extensible materials, and the non-extensible LB (REDIX-L350, Acetech, Seoul, Korea) was composed of non-extensible nylon. Given the variations in physical characteristics and sizes of participants, ready-made products of three different types (small, medium, and large) were used. The LB was worn such that the lower edge of the belt did not touch the thigh when seated and cover the anterior superior iliac spine of the pelvis [22]. The wearing pressure was standardized at 60 mmHg by measuring with a thin force-sensing resistor embedded between the LB and the right iliac crest of the participant in a standing position [36] (Figure 1).

### 2.3. Experimental Procedure

The temperature and surrounding environment of the experiment room were set for the participants to feel comfortable. To avoid any disturbance in measurement, the participants wore sleeveless shirts and shorts, which were light and form-fitting. Prior to measurement, the examiner conducted anthropometric measurements to assess the general characteristics of the participants.

**Figure 2:**
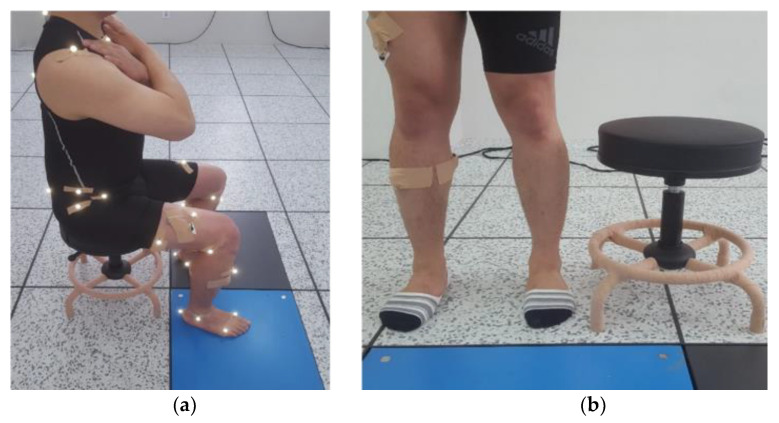
Experiment preparation; (**a**) initiating the standing-up position from the sitting position; (**b**) stool height.

After attaching the motion capture markers and preparing for the experiment, the participants were seated on an adjustable stool with their bare feet placed at a comfortable width on a force plate installed on the floor (Figure 2a). The height of the stool was adjusted at 90% of the height of the knee from the floor, which represents the distance from the floor to the fibular head (Figure 2b). The participants were seated at their preferable comfort posture and looked forward, with their arm crossed in front of their chests, without covering the motion capture markers to prevent any disturbance caused by arm swinging [37].

Following the verbal signal of research assistants operating computers connected to the data collection system, the participants stood at a pace they chose, stayed in a comfortable standing position for 3 s, and then sat on the stool again. A demo video was shown to the participants to make them understand the experimental procedure, and measurement was performed after practicing the procedure 2–3 times. The participants performed the STD movement in a manner in which they felt comfortable, with the only limitation of maintaining the positions of their feet. The initial locations of the feet were marked using a masking tape, and the STD movement was performed in the same location for all conditions.

**Figure 3:**
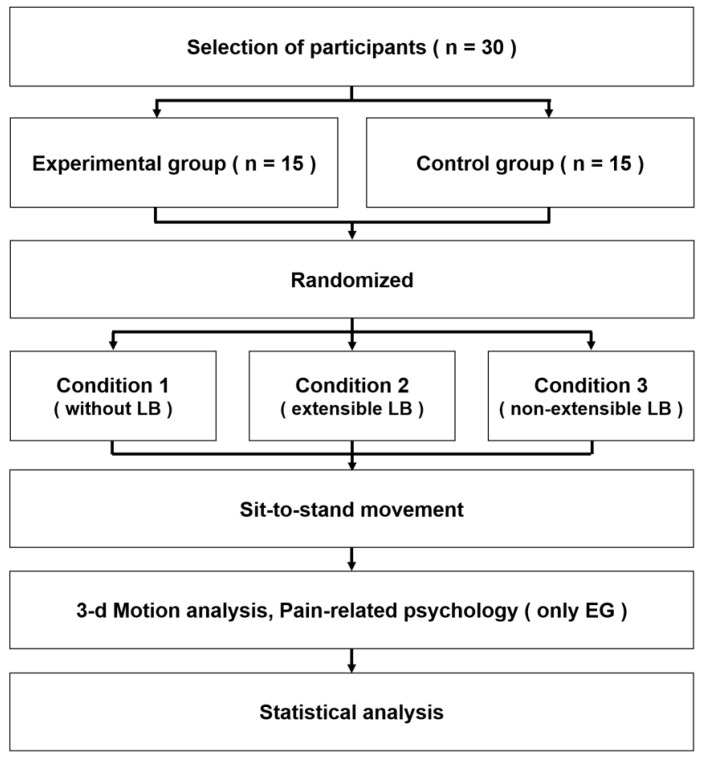
Diagram of experimental procedure.

There were three different experimental conditions: not wearing a LB (Condition 1), wearing an extensible LB (Condition 2), and wearing a non-extensible LB (Condition 3). To avoid the learning effect, the experimental conditions were set in a random order: participants picked up cards from a sealed envelope containing cards specifying the experimental conditions. Biomechanical variables of the STD movement in each condition were measured for all participants (Figure 3). To improve reliability, mean values of three successful measurements in each condition were analyzed, such that all participants performed the STD movement nine times. The measurement was considered successful when all the markers were visible, and the signals were properly recorded. A 1-min rest was given between each experiment, and a 5-minute break was given between each condition. Pain-related psychological variables were measured only in the experimental group immediately after completion of the STD movement in each condition.

### 2.4. Measurement Methods

#### 2.4.1. Measurement of Biomechanical Variables

To collect biomechanical data of physical movement of the participants while performing the STD movement, Qualisys Motion Capture System (Qualisys Medical AB, Gothenburg, Sweden) and force plate (Kistler, Winterthur, The Switzerland) were used. The resolution of Qualisys Motion Capture System was 1280 × 1024 pixels, and the sampling frequency was set at 200 Hz. Three-dimensional (3D) space coordinates were obtained using nonlinear transformation. The sampling frequency of the force plate was set at 2000 Hz. All biomechanical data were measured using Qualisys Track Manager (Qualisys Medical AB) software and was low-pass filtered using a fourth order Butterworth filter with cut-off frequencies of 25 Hz and 10 Hz. After that, the data were analyzed using Visual3D software (C-Motion, Germantown, Rockville, MD, USA) [38].

**Figure 4:**
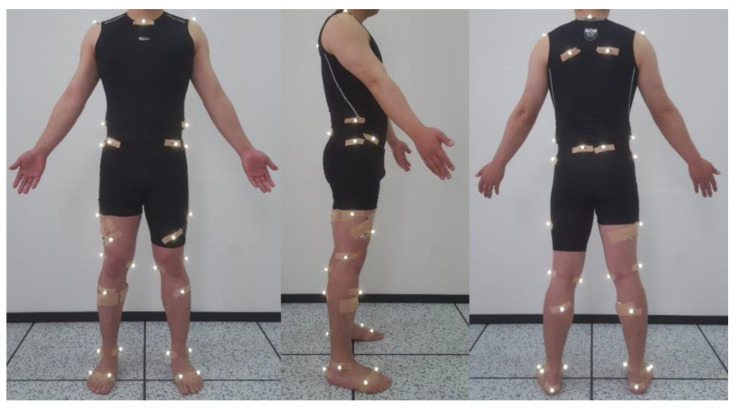
Location of the reflective motion capture markers.

Before implementing the test, calibration was performed to correct potential errors in the thermographic camera. An examiner attached 36 reflective markers on the participant’s skin at different locations on the body, referring to a previous study and modifying it for the purpose of this study [39] (Figure 4). A static test was performed before performing measurements in the three experimental conditions to check the location of all joints on the computer in a stationary standing position with bare feet. For this, the participants were instructed to stand still on the force plate for 2 s. To prevent swinging or detachment, the markers were fixed using a Kinesio tape, which is not reflective. After the static test, the length from the lateral malleolus of each ankle to the markers (anterior superior iliac spine, posterior superior iliac spine, and top of iliac crest) covered by the LB was measured in the standing upright position, and the distance between each marker was measured using a flexible ruler. Using this measured distance/length, the markers were reattached to the same location in other LB conditions.

#### 2.4.2. Measurement of Pain-Related Psychological Variables

Pain-related psychological variables were measured only in the experimental group in each condition. Immediately after the STD movement in each condition, participants were asked to recall the STD movement they just had completed by measuring pain intensity, pain-related anxiety, and pain catastrophizing. The psychological values of pain-related anxiety and pain catastrophizing were measured using a tool that can be adjusted to meet specific biological conditions in a laboratory, as suggested in a previous study [40].

Participants were asked to evaluate pain intensity on an 11-point scale immediately after performing the STD movement [34]. On this scale, 0 represents no pain and 10 represents extreme pain. Pain-related anxiety was assessed using the photograph series of daily activities (PHODA) scale [41]. This included asking and assessing the participants’ level of worry about aggravating waist pain with regard to the STD movement they had just completed using the following question: “People with pain worry that specific activities might worsen the pain. What is your level of worry regarding the STD movement possibly aggravating the pain?” The score was quantified using a 10-cm VAS from 0-point (“none”) to 10-point (“extremely worried”). Pain catastrophizing was measured by the number of positive answers to the pain catastrophizing questionnaire [40]. This measurement method, composed of 6 items, has shown good internal consistency (Cronbach’s alpha = 0.87) in a previous study [42]. However, since the duration of the STD movement is not enough to use a frequency scale, a binary method was used for the responses [31].

### 2.5. Data Processing

**Figure 5:**
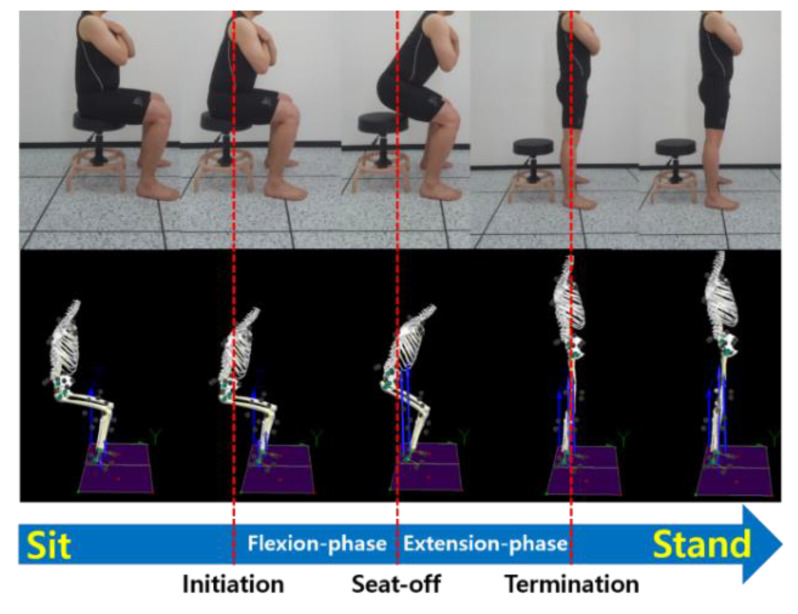
Indices and classification of the standing-up position from the sitting position.

To analyze the biomechanical data, the STD movement was classified into three timepoints and two phases (Figure 5). Using parameters of ground reaction force (GRF) measured from the force plate, three timepoints were decided. Initiation point [43] and seat-off point [44] were determined on the basis of the vertical GRF parameter, and the termination point [45] was determined on the basis of the anterior-posterior (A-P) GRF. The minimum and maximum values of the GRF data were used for calculating potential changes in the parameters of the STD movement. Initiation point was defined as a 5% change in threshold value from the baseline value of vertical GRF in a seated-still position, and termination point was defined as a 7.5% change in threshold value from the final value at A-P GRF standing-still position [46]. The seat-off point was set as the maximum value of vertical GRF [45]. Each phase was classified as follows: flexion phase (from initiation point to seat-off point); extension phase (from seat-off point to termination point) [47]. Each STD movement was normalized to 101 data points ranging from the initiation point to the termination point [33]. The maximum value obtained during the STD movement was used for joint angle and GRF. The values of flexion phase, extension phase, and total phase were used for the ROM.

### 2.6. Statistical Analysis

The data collected in this study were analyzed using SPSS 22.0 for windows (IBM Corp., Armonk, NY, USA). Normality of the data were tested using the Shapiro–Wilk test. General characteristics of the participants were analyzed using descriptive statistics, and homogeneity between the two groups was tested using independent *t*-test and Chi-square test.

Two-way repeated analyses of variance (ANOVA) (2 Group × 3 Conditions) were performed to evaluate the presence of lower back pain and changes depending on the conditions of wearing a LB. In cases where the interaction was significant, ANOVA was used for each group to determine the difference between the conditions. If there was a significant difference between conditions, a post hoc analysis was performed using an independent *t*-test for each condition. Differences between groups in each condition were confirmed using Bonferroni correction. Least square difference (LSD) was used for comparison of the main effect. In cases where both the interaction and main effects were significant, only interaction was analyzed. To identify pain-related psychological variables according to the conditions in the experimental group, one-way repeated ANOVA was performed, and LSD was used for post-hoc analysis. The level of statistical significance (α) was set at 0.05, and when the interaction was significant, the significance level of Bonferroni correction (α) was set a 0.017.

Cohen’s d formula was used for the effect size corresponding to the effect detected in the interaction, main effect, and post-hoc analysis [48]. The effect size was calculated using G-power 3.1.9.4. In the ANOVA, the effect size “*f*” was interpreted as “small” for 0.10, “medium” for 0.25, and “large” for 0.40. In the independent *t*-test, the effect size “*d*” was interpreted as “small” for 0.20, “medium” for 0.50, and “large” for 0.80 [49].

## 3. Results

### 3.1. Analysis of General Characteristics of the Experimental and Control Groups

General characteristics of the experimental and control groups are presented in Table 1. There was no significant difference in the general characteristics of participants between the two groups (*p* > 0.05).

### 3.2. Analysis of Biomechanical Variables According to the Conditions of Wearing a Lumbar Belt in the Experimental and Control Groups

**Figure 6:**
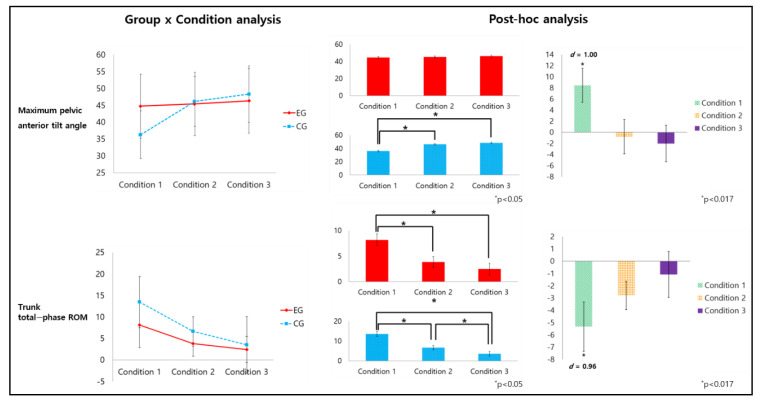
Interaction effects of maximum anterior pelvic tilt and total-phase ROM of the trunk.

There was an interaction effect in the maximum anterior pelvic tilt angle (*p* < 0.05, *f* = 0.79) (Table 2). From the post-hoc analysis, a significant difference was found (*p* < 0.017, *d* = 1.00) between the two groups in Condition 1 (without LB), but no significant difference was found (*p* > 0.017) in Conditions 2 and 3 (with LB) (Figure 6). There was an interaction effect in the trunk total-phase ROM (*p* < 0.05, *f* = 0.41) (Table 2). From the post-hoc analysis, a significant difference was found (*p* < 0.017, *d* = 0.96) between the two groups in Condition 1 (without LB), but no significant difference was found (*p* > 0.017) in Conditions 2 and 3 (with LB) (Figure 6).

There was a main effect of wearing a LB in the maximum trunk flexion angle, the maximum hip-joint flexion angle, and the maximum knee-joint flexion angle (*p* < 0.05, *f* = 0.38–0.87) (Table 2). Regarding maximum trunk flexion angle and maximum knee-joint flexion angle, significant differences were found between Conditions 1 and 2 and between Conditions 1 and 3 (*p* < 0.05). Regarding maximum hip-joint flexion angle, significant differences were found among all conditions (Conditions 1, 2, and 3) (*p* < 0.05).

There was a main effect of wearing a LB in trunk flexion phase and extension phase ROM (*p* < 0.05, *f* = 0.92, 2.35) (Table 2). With regard to trunk flexion-phase ROM, significant differences were found between Conditions 1 and 2 and between Conditions 1 and 3 (*p* < 0.05). Regarding trunk extension-phase ROM, significant differences were found among all conditions (Conditions 1, 2, and 3) (*p* < 0.05).

There was a main effect of wearing a LB in hip-joint flexion-phase, extension-phase, and total-phase ROM (*p* < 0.05, *f* = 0.55 to 1.12) (Table 2). Regarding the hip-joint flexion-phase ROM, significant differences were found between Conditions 1 and 2 and between Conditions 1 and 3 (*p* < 0.05). With regard to hip-joint extension-phase and total-phase ROM, significant differences were found among all conditions (Conditions 1, 2, and 3) (*p* < 0.05).

There was a main effect between the groups with regard to the knee-joint flexion-phase ROM (*p* < 0.05, *f* = 0.52) (Table 2), and a significant difference was found between the experimental and control groups (*p* < 0.05). There was also a main effect of wearing a LB in knee-joint extension-phase and total-phase ROM (*p* < 0.05, *f* = 0.41, 0.50) (Table 2). Significant differences were found between Conditions 1 and 2 and between Conditions 1 and 3 (*p* < 0.05).

There was a main effect of wearing a LB in anterior-posterior GRF and vertical GRF (*p* < 0.05, *f* = 0.38, 0.43) (Table 2). With regard to anterior-posterior GRF, there were significant differences between Conditions 1 and 2 and between Conditions 1 and 3 (*p* < 0.05). Further, there was a significant difference between Conditions 1 and 3 in vertical GRF (*p* < 0.05).

### 3.3. Analysis of Pain-Related Psychological Variables According to the Conditions of Wearing a Lumbar Belt in the Experimental Group

There were significant differences between the conditions of wearing LB in VAS, PHODA, and pain catastrophizing in the experimental group (*p* < 0.05, d = 0.83 to 2.00) (Table 3). From the post-hoc analysis results, there were significant differences between Conditions 1 and 2 and between Conditions 1 and 3 (*p* < 0.05) (Table 3).

## 4. Discussion

The purpose of this study was to investigate the effect of wearing extensible and non-extensible LB on biomechanical factors of the STD movement in office workers with LBP and on pain-related psychological factors. From the results obtained, an interaction effect between the presence of LBP and LB use was found in the biomechanical factors of the STD movement. In addition, a positive effect of wearing a LB was found on the pain-related psychological factors. Extensible and non-extensible LB had similar overall effects.

### 4.1. Analysis of Interactions between Groups and Conditions of Wearing a Lumbar Belt

An interaction effect was found between the ROM of the trunk and maximum anterior pelvic tilt angle in the STD movement. There was an interaction effect in the ROM of the total phase of the trunk. As a result of post-hoc analysis, trunk ROM in Condition 1 (without LB) was significantly smaller in the experimental group compared with that in the control group. Trunk ROM was decreased in Conditions 2 and 3 (with LB) compared with that in Condition 1 (without LB) in both experimental and control groups, with no significant difference between the two groups. Previous studies have reported that the lumbar ROM decreases in patients with LBP while performing the STD movement [50]. The trunk ROM was significantly lower in the experimental group in this study, which is consistent with the result of the previous study. With regard to the biomechanical factors, it was reported that continuously wearing a LB reduces the ROM of the trunk in various loads and movements [51,52]. Hence, it was considered that these effects of the LB reduced the ROM of the trunk in both the experimental and control groups while performing the STD movement. Given the large effect size and regardless of LBP, LB are effective in reducing the ROM of the trunk. In addition, there was an interaction effect in the maximum anterior pelvic tilt angle. According to the post-hoc analysis, in Condition 1 (without LB), the maximum anterior pelvic tilt angle was significantly larger in the experimental group compared with that in the control group. Considering the maximum trunk flexion angle, these differences in the maximum anterior pelvic tilt angle are thought to be protective in patients with LBP, which thereby reduce trunk flexion and maintain a rigid lumbar region. The maximum anterior pelvic tilt angle was increased only in the control group in Conditions 2 and 3 (with LB) compared with Condition 1 (without LB), with no significant difference between the two groups. In a previous study that analyzed the effect of a LB on the lumbar ROM and lumbopelvic rhythm, it was reported that wearing LB limits the lumbar ROM and alters the lumbopelvic rhythm [24]. In this study, a LB was worn to cover the pelvis such that the lower edge of the LB covered the ASIS and lilac crest. Therefore, the ROM of the trunk decreased in both groups, and it is thought that the rigidity resulting from wearing the LB affected the spine–pelvis junction. In STD movement, anterior driving power that accelerates the center of mass (COM) of the body is generated by trunk flexion [53]. Trunk ROM was decreased in the experimental group while wearing the LB, but it is thought that the anterior driving power that accelerates the COM of the body, which is necessary to perform the STD movement, was generated by the increased maximum trunk flexion angle. Although there was no change in the maximum trunk flexion angle in the control group while wearing LB, the trunk ROM was decreased. Therefore, the maximum anterior pelvic tilt angle was increased owing to the rigidity resulting from wearing a LB. Consequently, the decreased ROM of the trunk in Conditions 2 and 3 (with LB) compared with Condition 1 (without LB) in both groups and the increased maximum anterior pelvic tilt angle in the control group could be explained by the rigidity resulting from wearing LB. A large effect size was shown in the difference between the maximum anterior pelvic tilt angle and ROM of the trunk in the experimental and control groups in Condition 1 (without LB). Given the large effect size, it is thought that the difference between the maximum anterior pelvic tilt angel and the ROM of the trunk demonstrates that the STD movement is performed differently in the experimental and control groups.

### 4.2. Analysis of the Main Effect between the Two Groups

A main effect between the two groups was found in the ROM of knee-joint flexion phase of the STD movement. The knee-joint ROM was significantly increased in the experimental group compared with that in the control group. To perform the STD movement correctly, coordinated behavior of the lumbopelvic complex is required [33]. When performing the STD movement, a healthy individual completes the standing process by simultaneously bending the spine and hip joint and then simultaneously extending them [54]. In this study, it was found that the subjects from the control group performed the STD movement using the trunk and hip joint while the subjects from the experimental group used the knee joint more. Considering the large effect size, large ROM of the knee joint in flexion phase is one of the features of the STD movement in the experimental group. Although this is not statistically significant, the maximum flexion angle of the trunk in the experimental group was smaller during the STD movement than that in the control group. A previous study also reported decreased lumbar ROM and maximum flexion in patients with LBP [50]. In addition, the maximum anterior pelvic tilt angle and ROM of the hip joint were bigger in the experimental group compared with that in the control group, which is similar to a previous study that also reported increased ROM of the hip joint [55]. Likewise, subjects in the experimental group performed the STD movement with a rigid lumbar region by increasing anterior pelvic tilt angle and decreasing trunk flexion angle and ROM. This is thought be a result of a protective strategy undertaken by patients with LBP to avoid further stress on the pain area and reduce load to the posterior lumbar region by reducing the trunk flexion angle and movement. In a previous study, it was stated that the lower extremities are involved in possible compensation exercises in patients with LBP [56]. Further, in this study, it is thought that the STD movement was performed by increasing the flexion angle and ROM of the knee-joint to compensate for the decreased flexion angle and ROM of the trunk.

### 4.3. Analysis of the Main Effects of the Conditions of Wearing Lumbar Belts

The maximum trunk flexion angle of the STD movement increased significantly in Conditions 2 and 3 (with LB) compared with that in Condition 1 (without LB). Maximum flexion angle, extension-phase ROM, and total-phase ROM of the knee joint were significantly decreased. LB may decrease the stress on posterior viscoelastic structures or compression load on the lumbar region [25,26]. This may decrease the load on specific spinal structures as an effect of lumbar movement limitation due to increased mechanical rigidity caused by the LB [51,52]. Further, it is thought that the maximum trunk flexion angle was increased as a protective strategy to decrease the effect of the reduced trunk flexion angle in order to avoid stress in the pain area by wearing a LB. Therefore, the compensation effect that occurred in the knee joint also decreased.

The flexion-phase and extension-phase ranges of motion during the STD movement were significantly decreased in Conditions 2 and 3 (with LB) compared with that in Condition 1 (without LB). In addition, the maximum flexion angle and ROM of the hip joint were increased significantly. Several previous studies have reported decreased ROM of the trunk due to rigidity resulting from wearing a LB [57,58,59]. The same results were also observed in this study, wherein the ROM of the trunk was decreased in Conditions 2 and 3 (with LB). Considering the large effect size, it is thought that the LB is effective in decreasing the ROM of the trunk. In this study, the maximum trunk flexion angle was maintained despite wearing the LB. However, because of the rigidity resulting from wearing the LB, the spinopelvic area became rigid, thereby resulting in forward inclination of the pelvis and increased flexion angle and ROM of the hip joint.

### 4.4. Analysis of Pain-Related Psychological Variables According to Lumbar Belt Conditions

In the experimental group, positive effects of wearing a LB on pain-related psychological variables were found while performing the STD movement, with the two types of LB showing similar effects. Pain intensity measured by VAS was significantly decreased in Condition 1 (without LB) compared with that in Conditions 2 and 3 (with LB). The possible mechanisms of pain relief of the LB include decreased ROM [60,61], improved proprioceptive sense or motor sense [62,63], and strengthened trunk [51,60,64]. Results of this study are in accordance with those of a previous study that reported a significant decrease in pain after wearing a LB for an hour for three weeks [65]. However, as the decrease did not reach the 30% threshold, which is considered to be clinically important in the measurement of pain and disability [66], further scientific and systematic studies on the effects of wearing LB on pain intensity are warranted.

Pain-related anxiety and pain catastrophizing were significantly decreased in Condition 1 (without LB) compared with that in Conditions 2 and 3 (with LB). This is thought to be because of the mechanical support provided by the LB, which affects pain and pain-related psychology. According to previous studies, motivational situations from exposure-based treatment have an impact on long-term changes in fear of pain [67]. The decreased pain intensity, pain-related anxiety, and pain catastrophizing could reduce disabilities by increasing self-efficacy, which may lead to gradual exposure to labor at work and maintaining these activities at work [68]. Further, as per the biomechanical analysis, the LB benefits office workers in achieve this purpose. The hypothesis that the mechanical support provided by a LB may result in muscle weakness leaves some room for controversy [69]. However, according to the psychological studies, the LB does not induce a wrong compensatory sense [70]. According to a recent systemic review, long-term use of a LB does not necessarily result in the deterioration of trunk muscles [69]. Furthermore, intermittent use of a LB may reduce the probability of trunk muscle weakening [71]. In this study, the effect size of wearing a LB on pain-related psychological factors was large. Therefore, it is thought that wearing a LB may be psychologically helpful for office workers to gradually increase their activity, return to the workplace, or maintain a job.

### 4.5. Limitations

There are several limitations of this study. First, the number of subjects was relatively small. Second, only the immediate effect, not the long-term effect, of wearing a LB was investigated. Third, movement of each spinal segment or compensation effect of the thoracic vertebrae were not considered because the spine was investigated as a whole. Forth, there was a restriction on the height of the stool and the location of the feet since this study only focused on the effect of the LB on STD movement. Therefore, if the height of the stool and the location of the feet were not restricted, different results could have been obtained. Fifth, as there are various types and designs of LB, there may be differences in the obtained results depending on the differences in extensibility and design of the LB. Therefore, further studies with more subjects, different LB designs, biofeedback LB and using the placebo effect study methods are warranted.

## 5. Conclusions

Wearing an extensible or a non-extensible LB has beneficial effects on the biomechanical factors of the STD movement in both office workers with LBP and healthy office workers. This is attributed to reduced ROM of the trunk joint during the STD movement in office workers with LBP and healthy office workers. Moreover, wearing a LB engendered pain-related psychological advantages resulting in immediate reduction of pain intensity, pain-related anxiety, and pain catastrophizing in office workers with LBP. Extensible and non-extensible LB provide comfort and functionality suitable for use at work. Therefore, both LB would be helpful in the daily activities of patients with LBP, the laborious activities of office workers, and the return of office workers on leave due to LBP.

## Figures and Tables

**Table 1:** General characteristics of the experimental and control groups.

Variable	CG (*n* = 15)	EG (*n* = 15)	*p*
Sex (Male, %)	9 (60.0)	8 (53.3)	0.500 ^†^
Age (years)	34.53 (4.40)	35.60 (5.19)	0.549 ^‡^
Height (cm)	171.40 (9.83)	170.20 (8.08)	0.718 ^‡^
Weight (kg)	70.60 (12.50)	67.40 (14.60)	0.525 ^‡^
BMI (kg/m^2^)	23.85 (2.26)	23.02 (3.38)	0.437 ^‡^
VAS (score)		3.80 (0.86)	
ODI (%)		19.60 (4.42)	
Onset (month)		43.06 (33.35)	

* *p* < 0.05, ^†^ Chi-squared test, ^‡^ independent *t*-test. BMI: body mass index, CG: control group, EG: experimental group, ODI: Oswestry Disability Index, VAS: Visual Analog Scale.

**Table 2:** Analysis of biomechanical variables of the sit-to-stand movement according to the lumbar belt conditions in the experimental and control groups.

Variable	Condition 1 (No LB)		Condition 2 (Extensible LB)		Condition 3 (Non-Extensible LB)		*P (f)*			Post-Hoc
	CG	EG	CG	EG	CE	EG	Group (G)	Condition (C)	G x C	
Maximum trunk flexion (deg)	47.07 (10.33)	41.74 (7.26)	47.36 (10.86)	46.05 (6.21)	48.06 (9.47)	45.97 (7.01)	0.338	0.022 * (0.41)	0.100	C1 < C2, C1 < C3
Maximum anterior pelvic tilt (deg)	36.27 (7.10)	44.73 (9.58)	46.16 (8.40)	45.38 (9.57)	48.33 (7.39)	46.30 (9.43)	0.528	<0.001 * (0.99)	<0.001 * (0.79)	CG: C1 < C2, C1 < C3 C1: CG < EG
Maximum hip-joint flexion (deg)	104.21 (8.69)	107.13 (9.94)	111.81 (10.19)	112.14 (9.60)	115.04 (10.54)	112.89 (8.04)	0.939	<0.001 * (0.87)	0.162	C1 < C2, C1 < C3, C2 < C3
Maximum knee-joint flexion (deg)	77.93 (7.99)	83.40 (9.65)	76.43 (7.71)	81.25 (8.81)	76.16 (8.14)	81.78 (9.67)	0.095	0.019 * (0.38)	0.832	C1 > C2, C1 > C3
Maximum ankle-joint flexion (deg)	11.16 (5.84)	9.48 (3.70)	10.90 (5.47)	11.27 (5.77)	11.94 (4.83)	10.48 (6.80)	0.608	0.472	0.364	
Trunk flexion-phase ROM (deg)	7.48 (4.34)	4.55 (3.72)	1.65 (2.85)	0.76 (3.26)	1.14 (3.06)	0.54 (2.43)	0.056	<0.001 * (0.92)	0.315	C1 > C2, C1 > C3
Trunk extension-phase ROM (deg)	24.99 (8.42)	19.58 (5.85)	9.98 (7.56)	7.57 (5.22)	4.33 (3.13)	3.36 (2.50)	0.101	<0.001 * (2.35)	0.123	C1 > C2, C1 > C3, C2 > C3
Trunk total-phase ROM (deg)	13.47 (5.95)	8.14 (5.26)	6.65 (6.62)	3.85 (3.03)	3.54 (3.40)	2.46 (2.96)	0.052	<0.001 * (1.50)	0.016 * (0.41)	CG: C1 > C2, C1 > C3, C2 > C3 EG: C1 > C2, C1 > C3 C1: CG > EG
Hip-joint flexion-phase ROM (deg)	24.97 (6.43)	26.69 (6.46)	30.59 (5.93)	32.93 (6.55)	30.89 (6.05)	31.92 (5.31)	0.416	<0.001 * (1.12)	0.648	C1 < C2, C1 < C3
Hip-joint extension-phase ROM (deg)	99.90 (7.52)	105.27 (9.95)	109.62 (6.39)	110.00 (10.67)	110.48 (7.44)	113.92 (9.69)	0.279	<0.001 * (0.98)	0.202	C1 < C2, C1 < C3, C2 < C3
Hip-joint total-phase ROM (deg)	104.73 (8.69)	109.32 (7.39)	110.82 (8.09)	111.88 (10.39)	112.77 (6.95)	114.39 (9.45)	0.345	0.003 * (0.55)	0.452	C1 < C2, C1 < C3, C2 < C3
Knee-joint flexion-phase ROM (deg)	5.00 (1.99)	6.48 (2.04)	4.87 (1.94)	6.45 (2.04)	4.60 (2.33)	6.50 (2.10)	0.011 * (0.52)	0.896	0.857	EG > CG
Knee-joint extension-phase ROM (deg)	76.19 (10.88)	79.95 (11.94)	75.24 (12.32)	76.66 (10.59)	74.34 (9.71)	75.06 (9.96)	0.608	0.020 * (0.41)	0.357	C1 > C2, C1 > C3
Knee-joint total-phase ROM (deg)	80.72 (10.86)	84.64 (10.97)	76.21 (10.28)	82.32 (9.72)	77.51 (9.83)	80.85 (9.97)	0.220	0.002 * (0.50)	0.386	C1 > C2, C1 > C3
Ankle-joint flexion-phase ROM (deg)	6.57 (2.63)	6.86 (1.79)	6.45 (3.80)	5.58 (1.83)	6.88 (2.81)	5.68 (1.86)	0.473	0.211	0.150	
Ankle-joint extension-phase ROM (deg)	11.99 (3.48)	10.42 (3.10)	11.49 (4.89)	9.89 (3.69)	11.39 (4.91)	9.77 (4.02)	0.237	0.524	0.999	
Ankle-joint total-phase ROM (deg)	15.24 (4.66)	14.81 (4.26)	15.10 (4.62)	14.71 (3.85)	14.94 (4.55)	14.28 (3.25)	0.697	0.855	0.970	
Anterior-posterior GRF (N/kg)	−46.42 (12.41)	−41.85 (12.69)	−48.35 (10.48)	−47.78 (12.71)	−48.29 (13.26)	−48.87 (10.42)	0.701	0.040 * (0.38)	0.291	C1 < C2, C1 < C3
Medial-lateral GRF (N/kg)	68.58 (14.78)	63.34 (17.79)	69.11 (12.91)	63.54 (13.61)	66.76 (12.07)	61.50 (10.63)	0.262	0.386	0.994	
Vertical GRF (N/kg)	588.44 (38.37)	572.12 (31.94)	596.84 (29.14)	579.49 (45.78)	603.27 (43.55)	590.32 (30.24)	0.212	0.009 * (0.43)	0.906	C1 < C3

* *p* < 0.05; *f*, effect size “*f*”. CG: control group, EG: experimental group, ROM: range of motion, C1: Condition 1, C2: Condition 2, C3: Condition 3.

**Table 3:** Analysis of pain-related psychological variables in the experimental group.

Variable	Condition 1 (No LB)	Condition 2 (Extensible LB)	Condition 3 (Non-Extensible LB)	*p* (*f*)	Post-Hoc
VAS	4.07 (0.88)	3.27 (0.70)	3.47 (0.74)	0.001 * (0.83)	C1 > C2, C1 > C3
PHODA	4.27 (0.59)	3.20 (0.68)	3.40 (0.51)	<0.001 * (2.00)	C1 > C2, C1 > C3
PC	2.53 (0.64)	1.80 (0.41)	1.87 (0.35)	0.001 * (0.97)	C1 > C2, C1 > C3

* *p* < 0.05; *f*, effect size “*f*”. C1: Condition 1, C2: Condition 2, C3: Condition 3, PC: pain catastrophizing, PHODA: photograph series of daily activities, VAS: Visual Analog Scale.

## Data Availability

The data presented in this study are available on request from the corresponding author. The data are not publicly available due to ethical restrictions.
